# Rapid label-free detection of cholangiocarcinoma from human serum using Raman spectroscopy

**DOI:** 10.1371/journal.pone.0275362

**Published:** 2022-10-13

**Authors:** Peeraya Suksuratin, Rutchanee Rodpai, Vor Luvira, Pewpan M. Intapan, Wanchai Maleewong, Oranat Chuchuen

**Affiliations:** 1 Biomedical Engineering Program, Faculty of Engineering, Khon Kaen University, Khon Kaen, Thailand; 2 Department of Parasitology, Faculty of Medicine, Khon Kaen University, Khon Kaen, Thailand; 3 Mekong Health Science Research Institute, Khon Kaen University, Khon Kaen, Thailand; 4 Department of Surgery, Faculty of Medicine, Khon Kaen University, Khon Kaen, Thailand; 5 Department of Chemical Engineering, Faculty of Engineering, Khon Kaen University, Khon Kaen, Thailand; Accra Technical University, GHANA

## Abstract

Cholangiocarcinoma (CCA) is highly prevalent in the northeastern region of Thailand. Current diagnostic methods for CCA are often expensive, time-consuming, and require medical professionals. Thus, there is a need for a simple and low-cost CCA screening method. This work developed a rapid label-free technique by Raman spectroscopy combined with the multivariate statistical methods of principal component analysis and linear discriminant analysis (PCA-LDA), aiming to analyze and classify between CCA (n = 30) and healthy (n = 30) serum specimens. The model’s classification performance was validated using k-fold cross validation (k = 5). Serum levels of cholesterol (548, 700 cm^-1^), tryptophan (878 cm^-1^), and amide III (1248,1265 cm^-1^) were found to be statistically significantly higher in the CCA patients, whereas serum beta-carotene (1158, 1524 cm^-1^) levels were significantly lower. The peak heights of these identified Raman marker bands were input into an LDA model, achieving a cross-validated diagnostic sensitivity and specificity of 71.33% and 90.00% in distinguishing the CCA from healthy specimens. The PCA-LDA technique provided a higher cross-validated sensitivity and specificity of 86.67% and 96.67%. To conclude, this work demonstrated the feasibility of using Raman spectroscopy combined with PCA-LDA as a helpful tool for cholangiocarcinoma serum-based screening.

## Introduction

The epidemiology of cholangiocarcinoma (CCA) varies greatly in different world regions but mostly in northeastern Thailand. According to a report of the European Network for the cholangiocarcinoma study (ENS-CCA), the statistics revealed that the northeast of Thailand possessed the highest worldwide incidence of approximately 85 per 100,000 people per year [1]. CCA was more prevalent in males than females in this region (over 100 vs. 40 per 100,000 people) [2]. CCAs are commonly found in elderly adults over 65 ages [3]. According to data on CCA incidence from 1989–2018, the average age of CCA patients was 62.7±11.1 in males and 64.7±11.3 in females [4]. An infection with fish-borne liver flukes of the Opisthorchiidae family has been a major cause of CCA in Southeast Asia [2], leading to chronic inflammation in the bile duct such as (i) mechanical damage from a sucker, (ii) toxic excretory-secretion, and (iii) immunopathology [5]. As definite cancer causes, these helminths are classified by the International Agency for Research on Cancer (IARC) as Group 1 biological carcinogens (i.e., fluke-related CCA) [6]. However, in other regions where the liver flukes are not endemic (e.g., Europe, America, Australia), non-fluke-related CCA has also been reported [2]. CCA patients rarely display symptoms early, so most patients visit doctors when symptoms appear in stages 3 or 4 [7]. There are a variety of diagnostic techniques for CCA, such as ultrasonography [8], magnetic resonance imaging (MRI) [8], computed tomography (CT) [9], endoscopic retrograde cholangiopancreatography (ERCP) [10], and brush biopsy [11]. However, these diagnostic techniques are relatively costly, time-consuming, and can cause pain and discomfort to the patients. Current blood-test screening techniques also have limitations as they rely on the detection of the biomarkers (e.g., carbohydrate antigen (CA19–9), carcinoembryonic antigen (CEA)), which are nonspecific to CCA, leading to insufficient diagnostic sensitivity and specificity [12]. Thus, there has not yet been any specific and effective blood test currently available for CCA screening in a large population.

Raman Spectroscopy is a low-cost, label-free, rapid, and simple-used technique for detecting small-molecule analytes and biomarkers. It has been increasingly used in biomedical and clinical diagnostics. This spectroscopic technique relies on the vibration of molecules based on inelastic Raman scattering that emerges from the changing of photon energy levels, providing unique Raman fingerprints of the molecules. Therefore, it has been employed to detect and quantitatively measure small-molecule drugs in various pharmaceutical and biological matrices [13–15]. Raman spectroscopy can also identify biomolecules such as proteins, nucleic acids, lipids, and biomarkers, which are important for detecting the biochemical alterations in cells, tissues, or biofluids of various diseases [16–18]. It has been used to investigate and identify microbial phenotypic changes, such as detecting phenotypic differences in Escherichia coli enriched for 1-butanol tolerance [19], developing phenotypic profiles of Escherichia coli for antibiotic drug development research [20], and isolating carotenoid-containing cells using single-cell genomics based on Raman sorting [21]. In cancer research, Raman spectroscopy has been extensively studied for cancer detection in serum [22, 23], plasma [24, 25], and tissue [26, 27] for several cancer types, including gastric cancer [22], colorectal cancer [28], breast cancer [29], cervical cancer [30], prostate cancer [31] and laryngeal cancer [32]. However, the Raman signal is intrinsically weak due to the small Raman scattering cross-section of biological molecules (around 10^−30^ cm^-2^ per molecule) [33]. Surface-enhanced Raman scattering (SERS) has been developed by absorbing molecules onto metal nanostructure surfaces (e.g., silver and gold nanoparticles) to enhance the Raman signal [34]. Although the SERS technique can effectively amplify the Raman signal by up to 1013–10^15^ times [35], it requires relatively complicated sample preparation and often gives poor measurement repeatability. The electroporation-assisted surface-enhanced Raman spectroscopy (SERS) technique was developed for screening intrahepatic cholangiocarcinoma from a cell suspension mixed with silver colloids. The principal component analysis and linear discriminant analysis (PCA-LDA) technique was then employed to discriminate the intrahepatic cholangiocarcinoma cells (ICC) from the normal cells, achieving a sensitivity of 97% and a specificity of 96% [36]. However, at present, there has not been any study employing the Raman technique for cholangiocarcinoma screening with biofluid.

This study applied Raman spectroscopy to distinguish cholangiocarcinoma from healthy serum specimens by analyzing important biomolecular components and identifying major Raman marker bands for separating the two groups. Raman spectroscopy was also combined with PCA-LDA to analyze and classify Raman spectra of the serum samples. The model’s classification efficiency was validated using k-fold cross-validation (k = 5). This simple and low-cost technique can provide initial CCA screening using patient serum specimens. Besides, it is a minimally invasive diagnosis, which reduces the risks to the patients and allows for faster diagnosis. This work highlights the potential of Raman Spectroscopy in the biomedical field, demonstrating that the identified Raman marker bands and the Raman-combined PCA-LDA technique could be effectively used for cholangiocarcinoma detection and screening.

## Materials and methods

### Serum sample preparation

The study reported a retrospective study of medical records and obtained the residual CCA sera (leftover samples) from the routine investigation of thirty CCA (n = 30) volunteers before surgery. The sample size was calculated using Cochran’s formula:

$$Sample\;size\;(n)=\frac{z^{2}p(1-p)}{d^{2}}$$

[37].

A population proportion (p) of 0.00085 was used based on the incidence rate of cholangiocarcinoma in Thailand’s northeastern region, which was 85 per 100,000 individuals per year [38]. With a 95% confidence interval (95%CI, z = 1.96) and a 1.2% error margin (d = 0.012), the sample size was estimated to be 22.66. As a result, 30 specimens were collected for each study group.

The CCA group consisted of 7 serum specimens in the early cancer stages (stages T0, T1-2) and 23 in the advanced stages (stages T3-4). The group comprised all CCA types classified according to their anatomical location, i.e., intrahepatic, perihilar, and distal CCA. All healthy serum samples (n = 30) were randomly selected from a frozen biobank at the Department of Parasitology, Faculty of Medicine, Khon Kaen University. The average age of the CCA and healthy groups was 61 ± 9.45 and 64 ± 7.25 years old, respectively. All data were fully anonymized, and the IRB waived the requirement for informed consent.

Each CCA serum was separated from the venipuncture blood sample by centrifugation (Cobas 8100, Roche Diagnostics, Manheim, Germany) at 5,000 rpm (2,136 g) for 10 min. The serum samples were aliquoted into seven separated tubes (4 μl in each) and stored at -20°C for further Raman spectroscopic analysis. The study protocol was approved by the Khon Kaen University Ethics Committee for Human Research (HE641114).

### Confocal Raman measurements

**Fig 1:**
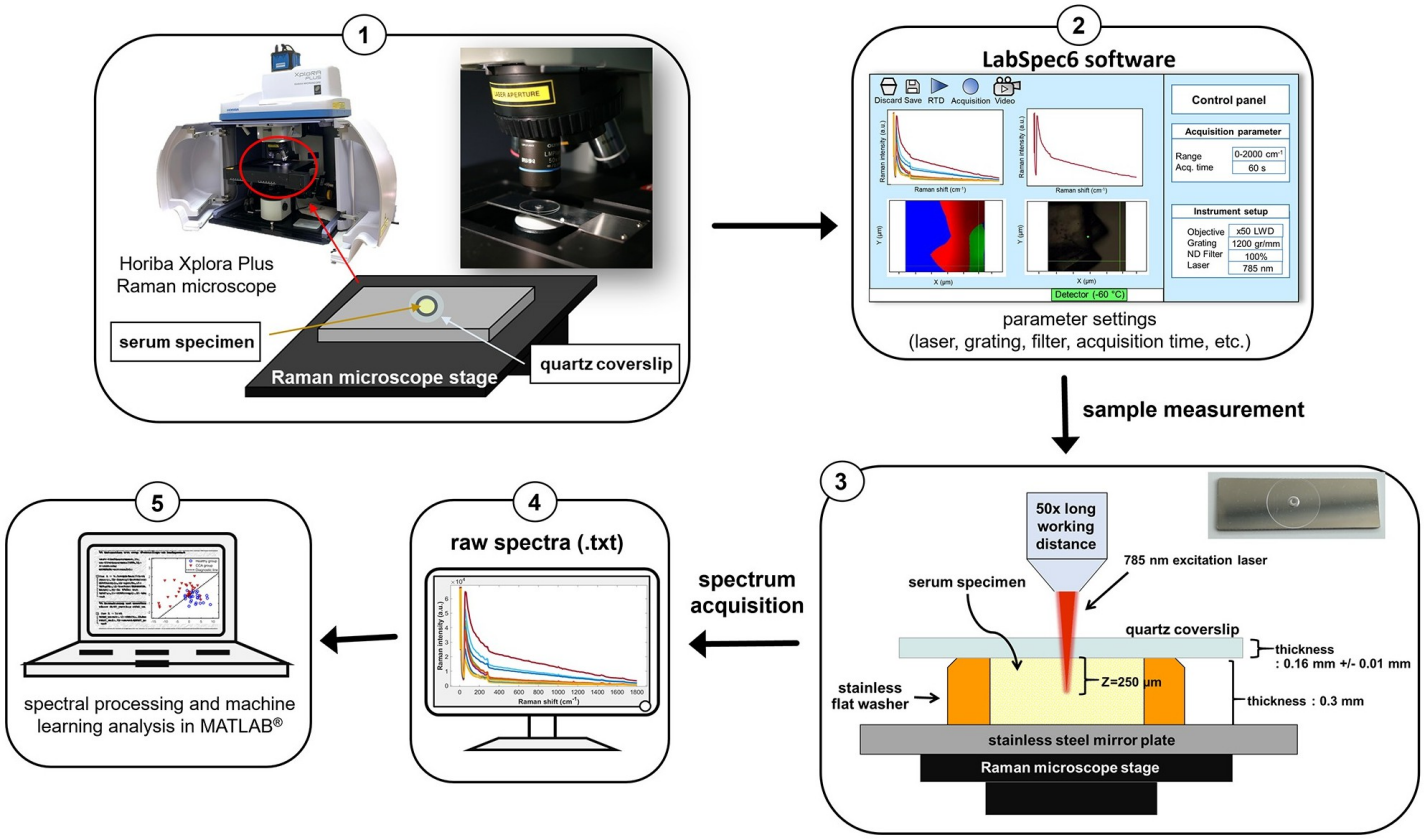
Experimental setup and procedure for Raman measurements of serum specimens of cholangiocarcinoma and healthy groups. The experimental steps consisted of: (1) placing a sample well containing 2.3 μl serum sample on a Raman microscope stage, (2) setting measurement parameters, (3) measuring serum samples with a laser excitation wavelength of 785 nm, (4) recording raw spectral data in LabSpac6 software, and (5) exporting the collected data to MATLAB for furfure spectral processing and machine learning classification analysis.

The serum of 2.3 μl was dropped onto a sample well made by attaching a flat washer (0.3 mm thickness, 2.8 mm diameter) onto a mirror-grade stainless steel plate. The sample well was covered with a quartz coverslip (R52500, Esco Optics, Oak Ridge, NJ) to prevent serum evaporation. The serum samples were measured with a Horiba XploRA PLUS confocal Raman microscope (Horiba Jobin Yvon, Northampton, UK) with a 50x objective lens (LMPLFL50X, Olympus, St. Joseph, MI) to provide a relatively stable signal in an integrated high-performance system. This instrument used a charge-coupled device (CCD) detector of the thermoelectric (TE) cooling type, which operated at -60°C to eliminate thermally-induced noise signals. It employed a 785 nm laser excitation, delivering a maximum output power of up to 90–100 mW. The near-infrared (NIR) laser light was used to reduce the background fluorescence interference and prevent sample damage from overheating. A grating of 1200 gr/mm, a hole diameter of 500 μm, and a slit of 200 μm width were used. Raman spectral data were recorded in.txt format by LabSpec 6 software (Horiba Scientific, Edison, NJ). Before each spectral measurement, the Raman microscope was calibrated with a silicon wafer for its Raman peak at 520 cm^-1^. Each serum sample was measured independently five times using an integration time of 60 seconds, collected in a spectral region of 0–2000 cm^-1^. A schematic summary of the overall experimental procedure used in this study is displayed in Fig 1.

### Raman data processing and peak height analysis

After the Raman spectra were recorded, initial spectral pre-processing and analysis were performed in Labspec 6. The spectral baseline of each spectrum was first subtracted by a polynomial fitting algorithm (degree 4). Next, each spectrum was smoothed using a Savitzky-Golay filter (20 points) and fitted with a Gaussian–Lorentzian function by a nonlinear least-squares method to calculate its peak height values. Statistical analysis was performed using IBM SPSS Statistics 26 (SPSS Inc., Chicago, IL) by an independent sample t-test with p-values of less than 0.05 considered statistically significant. Raman marker bands were selected from the peaks that exhibited a statistically significant difference in the peak height between healthy and CCA samples. The heights of these Raman bands were then input into a linear discriminant analysis (LDA) model for further classification analysis.

### Multivariate Raman spectral analysis

Raw Raman spectral data were exported to MATLAB software (R2021a, MathWorks, Natick, MA) for multivariate statistical analysis. Each Raman spectrum in the range of 350–1800 cm^-1^ was cropped for use in the analysis before being baseline-subtracted by a polynomial fitting algorithm (degree 4) and smoothed with a Savitzky-Golay filter (20 points). Next, the mean spectral intensity across all wavenumbers was used to normalize each spectrum. The processed Raman spectra were then analyzed by principal component analysis (PCA), which was used to reduce the variability of high-dimensional data by reducing the data features and transforming the spectral data into new variables called principal components (PCs). The scores of all PCs were analyzed by an independent sample t-test to identify the PCs that could statistically significantly distinguish between the CCA and healthy groups (p-value ≤ 0.05). The scores of the first ten PCs were input into the LDA model in MATLAB for human serum classification. The peak heights of the Raman marker bands obtained previously were also input into the LDA model. To prevent model overfitting, the Classification Learner App in MATLAB was employed for performing a cross validation analysis with k-fold cross-validation (k = 5). With this method, the data were randomly divided into k subgroups. The holdout approach was then repeated k times, with one of the k subsets providing the test set (validation set) and the other k-1 subsets forming a training set each time [39]. The performance estimation was averaged over all k trials to evaluate the model’s overall efficiency. The cross-validated results of the peak height-LDA and PCA-LDA methods, including the confusion matrices and the receiver operating characteristic (ROC) curves, were compared.

## Results and discussion

**Fig 2:**
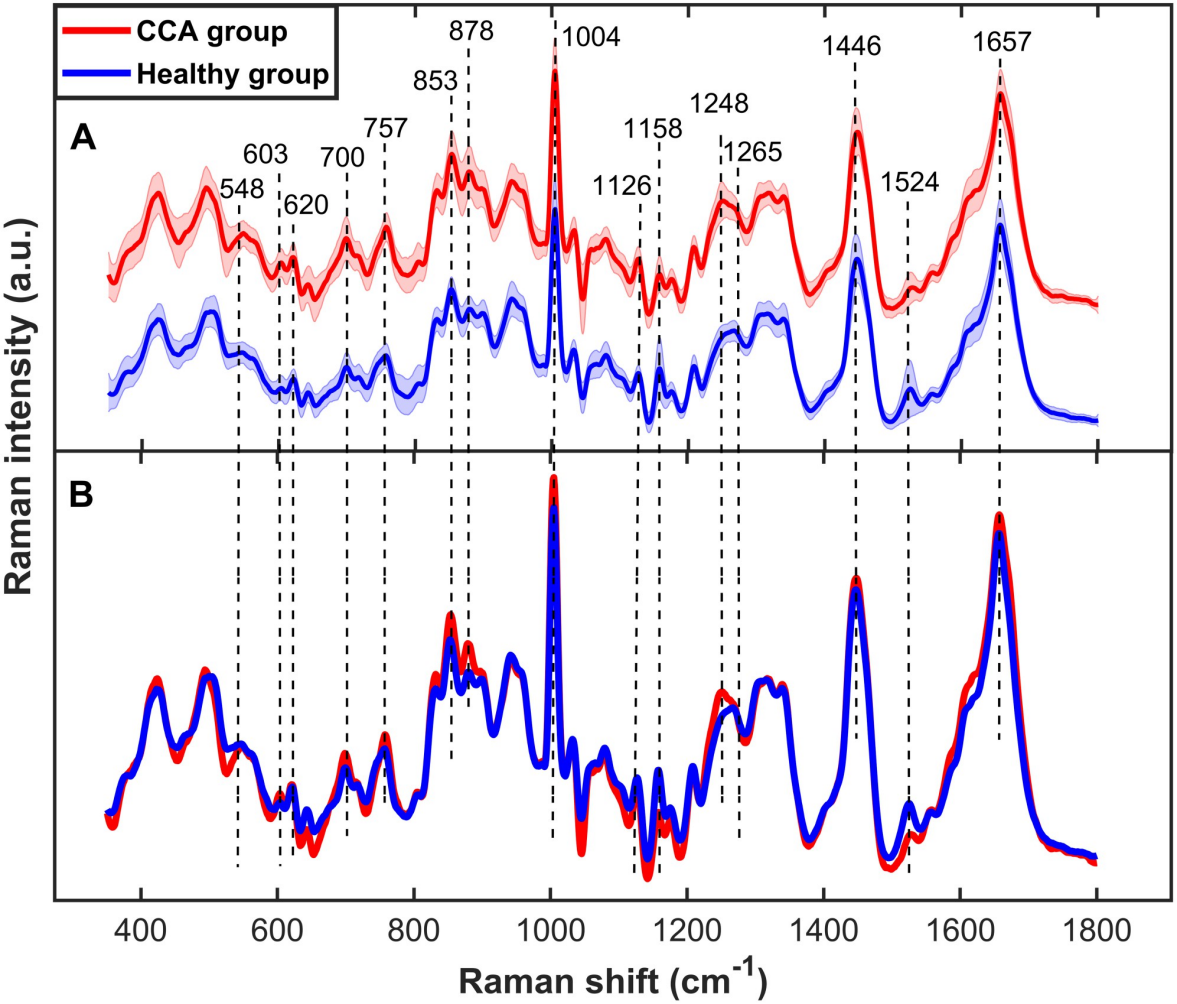
Mean serum Raman spectra of cholangiocarcinoma patients (red, n = 30) and healthy subjects (blue, n = 30). (A) Raman spectra that were offset for clarity of presentation. The solid lines and shaded regions show the averages and standard deviations of the data, respectively. (B) Superimposed average Raman spectra of the two groups.

**Fig 3:**
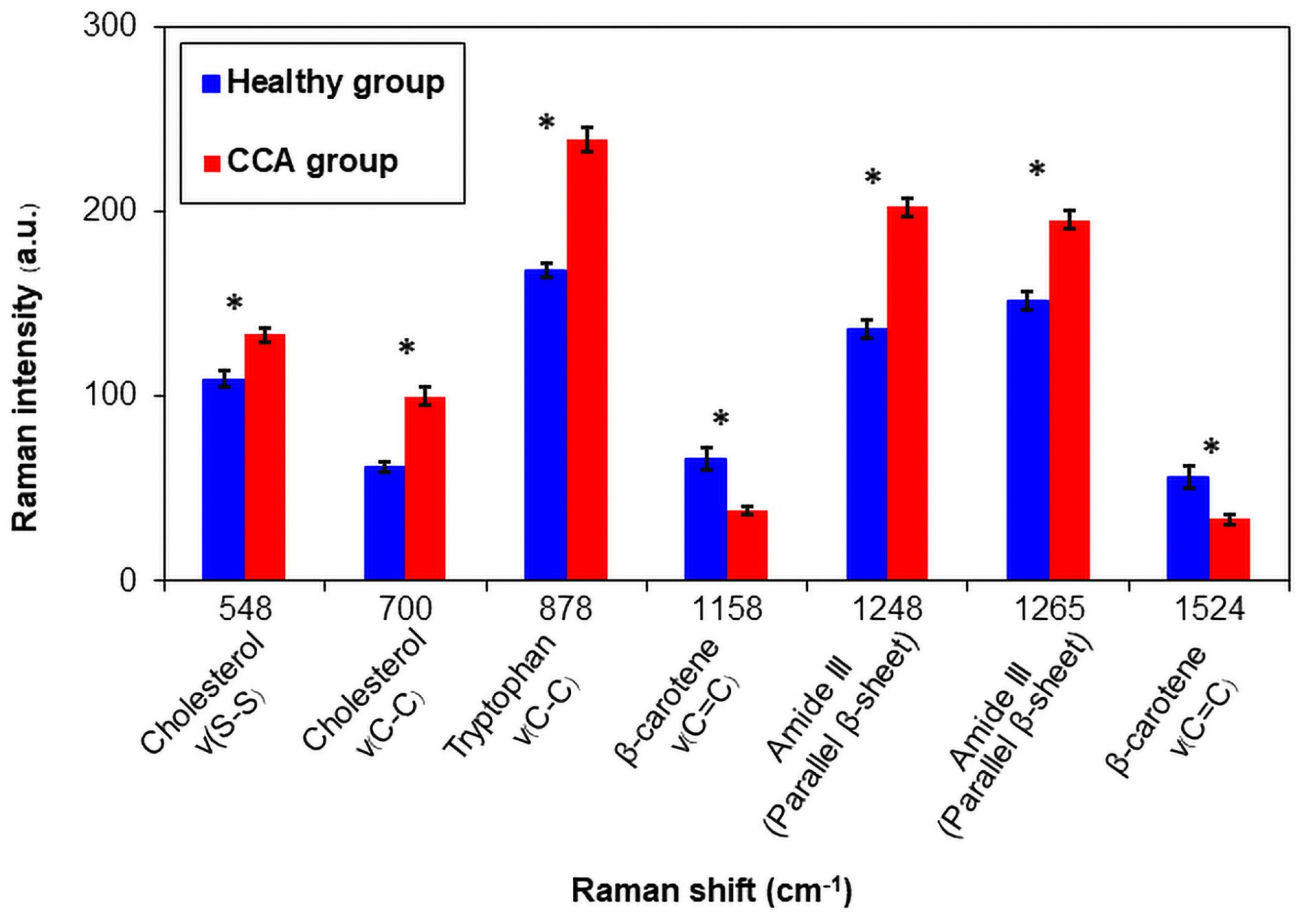
Comparison of averaged Raman peak intensities between the CCA and healthy groups. The Raman peak height values were calculated using a Gaussian–Lorentzian function. Data shown represent mean ± standard error of the measurements (150 total measurements for each group). Significant differences (p-value ≤ 0.05) are indicated by *. ν: stretching mode.

Each serum specimen from the cholangiocarcinoma (CCA) patients (n = 30) and the healthy subjects (n = 30) was measured independently five times using Raman spectroscopy. The mean serum Raman spectra of both subject groups and their biochemical element assignments are shown in Fig 2 and Table 1, respectively. The averaged peak intensities of the major Raman bands are displayed in Fig 3. All of these peaks exhibited a statistically significant difference in peak height between healthy and CCA groups (p-value < 0.05) and were selected as Raman marker bands to differentiate CCA samples from healthy ones. Interestingly, the serum beta-carotene peak intensities (1158, 1524 cm^-1^) of the healthy group were significantly higher than those of the CCA patients. On the contrary, the serum specimens of the CCA patients demonstrated significantly higher intensities in the Raman peaks of cholesterol (548 cm^-1^ and 700 cm^-1^), methionine (700 cm^-1^), tryptophan (757 cm^-1^ and 878 cm^-1^), and amide III (1248–1265 cm^-1^). These Raman maker peaks can be employed to distinguish the CCA from healthy subjects, and their peak heights can be combined with the LDA technique to improve the classification results.

**Table 1:** Peak positions and assignments of major Raman spectral peaks observed in serum of cholangiocarcinoma patients.

Raman shift (cm^-1^)	Band assignment	References
548	Cholesterol, S-S stretching mode	[40, 41]
603–620	C-C twist aromatic ring of phenylalanine	[40]
700	Cholesterol (C-S trans stretching mode), methionine	[29, 40, 41]
757	C-C stretching mode of tryptophan	[24, 35, 40–42]
853	Ring breathing mode of tyrosine, C–C stretch of proline ring	[41, 43–45]
878	C-C stretching mode of tryptophan	[24, 35, 40–42]
1004	C─C symmetric ring breathing mode of phenylalanine	[16, 22, 35, 40]
1126	Phospholipid, lipoproteins, C-C skeletal of acyl backbone in lipid	[41–43]
1158	C-C stretching mode of beta-carotene	[16, 22, 42]
1248–1265	Amide III, parallel beta-sheet	[23, 24, 35, 41, 43]
1446	CH_2_ bending mode of proteins, lipids, and some amino acid	[16, 24, 41, 42]
1524	C = C stretching mode of beta-carotene	[16, 22, 42]
1675	C = O stretching mode, amide I, α-helix	[25, 34, 35]

The differences observed in the serum biomolecular level of the CCA patients compared to the healthy group were consistent with the literature. Several studies found a correlation between the level of beta-carotene and the severity of cancer. Because beta-carotene is an antioxidant, it can inhibit cancer cell transformation at the molecular level by decreasing the growth rate and inducing apoptosis of the cancer cells [43, 46]. Our findings suggested that the CCA patients had lower levels of beta-carotene than the healthy group, agreeing with previous studies involving liver cancer [47], colorectal cancer [28], and nasopharyngeal cancer [48].

Furthermore, the Raman peaks of cholesterol at 548 cm^-1^ and 700 cm^-1^, whose intensities were higher in the CCA patients, could indicate a potential elevated serum cholesterol level resulting from the obstruction of the bile flow (cholestasis). Cholesterol is a precursor of bile acids. Therefore, the obstruction of the bile duct in CCA patients can cause a reduction in bile creation, leading to increased serum cholesterol levels [49]. Cholestasis is one of the risk factors of cholangiocarcinoma. It causes the accumulation of abnormal bile acids and leads to abnormal cell proliferation and genetic mutations of cancer cells [50]. Cholesterol has thus been selected as an indicator of necrosis in certain cancer cells, such those in the lung and liver [21, 51], in order to determine the progression from benign to malignant states and the severity of the disease [21]. It is consistent with our findings, in which the serum of the CCA patients demonstrated higher cholesterol peak heights (548 cm^-1^ and 700 cm^-1^) than the healthy group. The Raman peak found at 700 cm^-1^ could also be assigned to methionine, an essential amino acid potentially necessary for the growth of cancer cells that require exogenous methionine for survival and proliferation [52, 53]. Our findings showed that the Raman peak height of methionine was significantly higher in the serum specimens of the CCA patients. The growth of prostate, breast, and colorectal cancer cells has also been shown to be dependent on methionine [52].

In addition, the amide III Raman band (1248–1265 cm^-1^) was significantly higher in intensity in the CCA patients. It can be caused by the protein’s endogenous composition changes during metastasis or the increasing of the DNA and RNA expression caused by apoptosis or necrosis of the cancer cells [54]. When cells are degraded, intracellular elements such as nucleic acid fragments are released into the bloodstream, becoming cell-free DNA [54]. The RNA expression then stimulates increased protein production, resulting in a higher amino acid level. Our results were consistent with a previous study that intrahepatic cholangiocarcinoma cells (ICC) had abnormal DNA amplification ability leading to the higher nucleic acid level in ICC than the normal liver cells [36].

Moreover, the results suggested that the serum of the CCA patients contained comparably higher levels of tryptophan (757 cm^-1^ and 878 cm^-1^), agreeing with previous studies being reported for colon cancer [55], cervical cancer [43], and nasopharyngeal cancer [48]. Tryptophan plays a major role in protein synthesis and the generation of molecules that influence immunological activity [40]. Consequently, the alterations in the tryptophan metabolism and the enzyme indoleamine 2,3-dioxygenase activity may contribute to the changes in physiopathological conditions of cancer cells, acting as a promoter of cancer growth [48]. The study revealed that cancer cells exhibited important metabolic disorders, which presented an opportunity for spectroscopic techniques such as Raman spectroscopy to be used to detect these biomolecular changes. Raman spectroscopy can also be combined with machine learning techniques to more precisely identify and classify those spectral differences between the diseased and healthy groups.

To improve the efficiency of the CCA classification, the peak heights of the Raman marker bands, as identified in Fig 3, were input into a discriminant function LDA, a supervised machine-learning approach, to increase the variance between the two sample groups and decrease the variance within each group. In addition, the multivariate statistical method of PCA-LDA was employed. The PCA analysis, a dimensionality reduction method, transformed the Raman spectra features into independent variables known as principal components (PCs) before feeding them into the LDA model. Two sets of data were analyzed. (i) a total data set containing 300 measurements acquired from independently measuring each of the total 60 specimens (30 specimens from each group) five times; and (ii) an averaged data set acquired from averaging those five independent measurements of each specimen. The PCA analysis revealed six statistically significant PCs (PC-1, PC-2, PC-5, PC-8, PC-9, PC-12) from the total data set and three significant PCs (PC-1, PC-5, PC-7) from the averaged data set. As shown by the significant PC loadings in S1 Fig, the Raman spectral features of the CCA and healthy groups were clearly distinct and contained important spectral information that could be used to distinguish the two groups.

**Fig 4:**
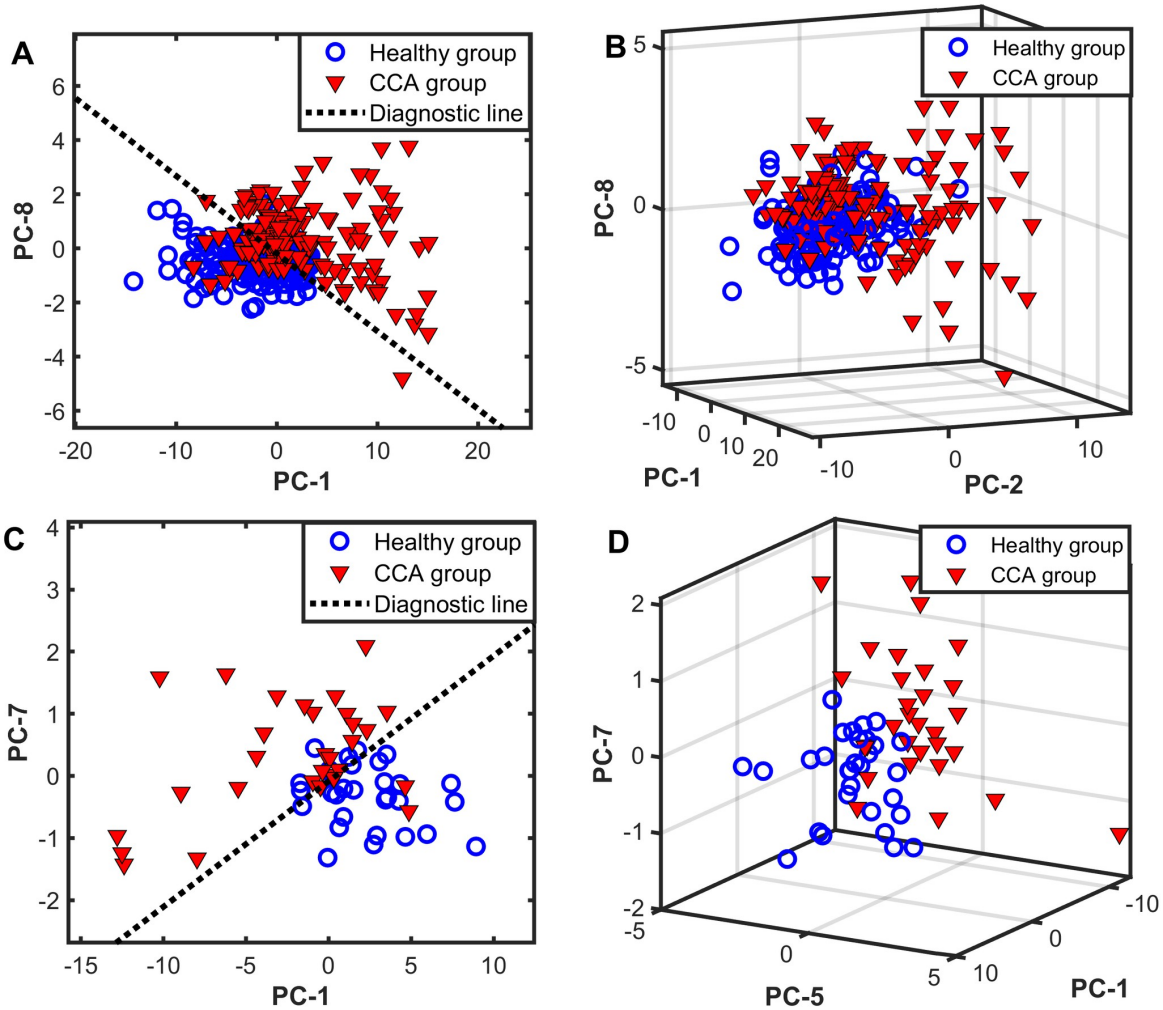
PCA score scatter plots of the healthy group (n = 30, blue) versus cholangiocarcinoma patients (n = 30, red). Before the PCA analysis, each Raman spectrum between 350 and 1800 cm-1 was baseline-subtracted and normalized. A and B panels display a total data set of 150 measurements for each group (30 specimens, each measured independently five times). C and D panels display an averaged data set obtained from averaging the five independent measurements of each specimen. The dotted line is a diagnostic line separating the two groups: (A) PC8 = -0.28PC1-0.19 and (C) PC7 = 0.20PC1-0.09.

The PCA score scatter plots revealed the two distinguishable clusters representing each subject group (Fig 4). The healthy group appeared to be more tightly grouped, while the CCA group was more dispersed and less clustered. A higher level of data dispersion of the CCA group could be due to the variations in the relative amounts of the biomolecular components due to the heterogeneous metabolic processes among different cancer patients. Moreover, using the averaged data set for the PCA analysis (Fig 4C) demonstrated superior diagnostic performance (sensitivity: 90.00%, specificity: 76.67%) than using the total data set (Fig 4A, sensitivity: 81.33%, specificity: 73.33%). Utilizing the average data set could improve the classification performance by reducing the intra-individual variability of human serum, which is a biological matrix with heterogeneous components.

**Fig 5:**
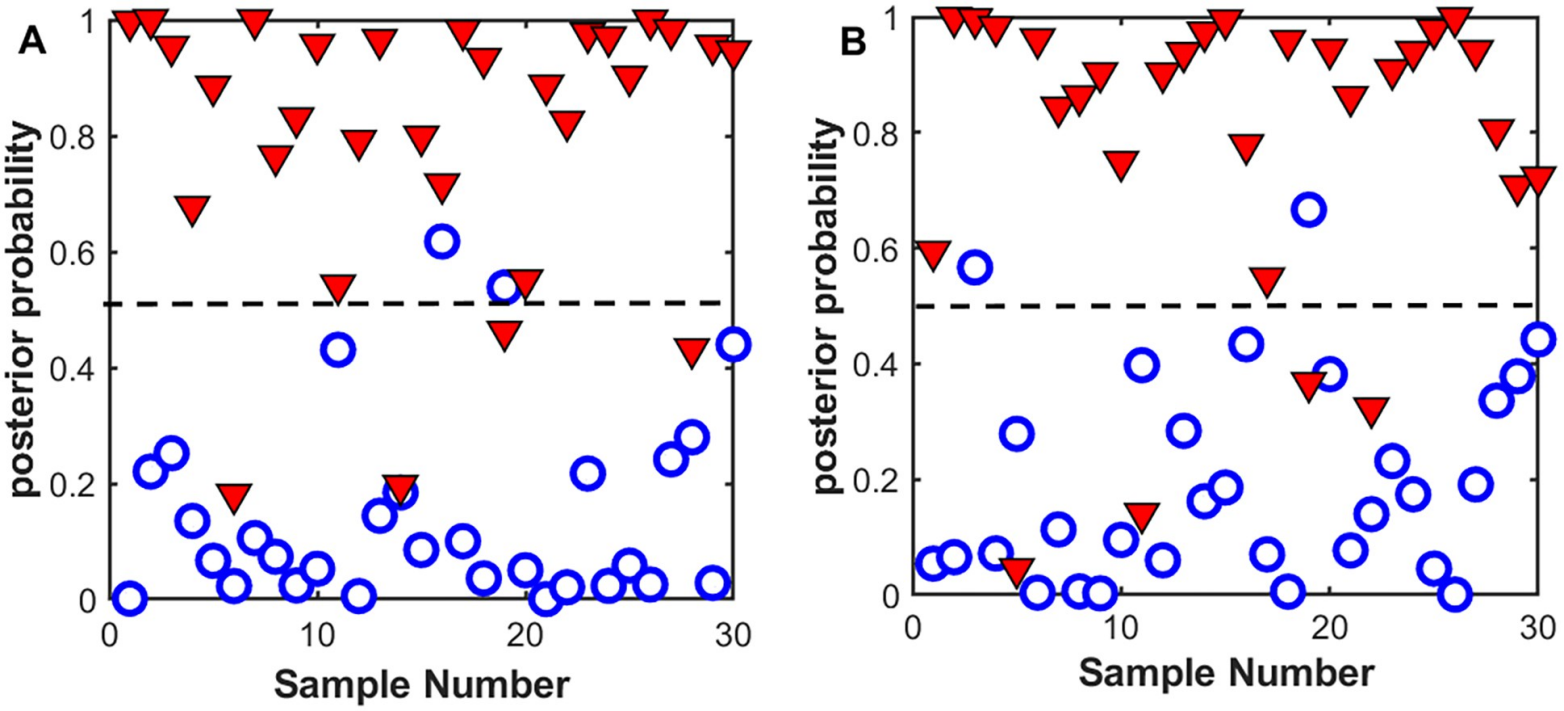
Scatter plots of the posterior probability of belonging to cholangiocarcinoma (n = 30) and healthy groups (n = 30). The posterior probability results were acquired by (A) the peak height analysis of the marker bands combined with LDA and (B) the PCA-LDA algorithm utilizing the first 10 PCs. The dotted line represented the discrimination threshold of 0.5.

To further improve the diagnostic classification between the CCA and healthy groups. All the first ten PCs of the averaged data set were loaded into an LDA model. The first ten PCs accounted for 94.56% of the total Raman spectral variance (PC-1: 40.16%, PC-2: 23.97%, PC-3: 11.06%, PC-4: 9.41%, PC-5: 4.21%, PC-6: 2.23%, PC-7: 1.13%, PC-8: 1.03%, PC-9: 0.75% and PC-10: 0.59%). For the peak height -LDA analysis, the peak heights of the seven Raman marker bands identified previously (Fig 3; 548, 700, 878, 1158, 1248, 1265 and 1524 cm^-1^) were input into the LDA model. Fig 5 shows the posterior probability of belonging to the CCA and healthy groups by a discrimination threshold of 0.5. The PCA-LDA approach demonstrated superior diagnostic performance (90% sensitivity and 100% specificity) to peak height-LDA (sensitivity: 86.67%, specificity: 90%). Notably, the peak height-LDA employed only the identified seven Raman marker bands (Fig 3) in the classification analysis. PCA-LDA, on the other hand, utilized ten PCs that together covered 94.56% of the total Raman spectral variance, and each PC contained a number of significant Raman spectral features extracted from the PCA analysis that could effectively differentiate the two groups. Thus, the combination of these PCs with LDA resulted in improvement of the overall classification performance.

**Fig 6:**
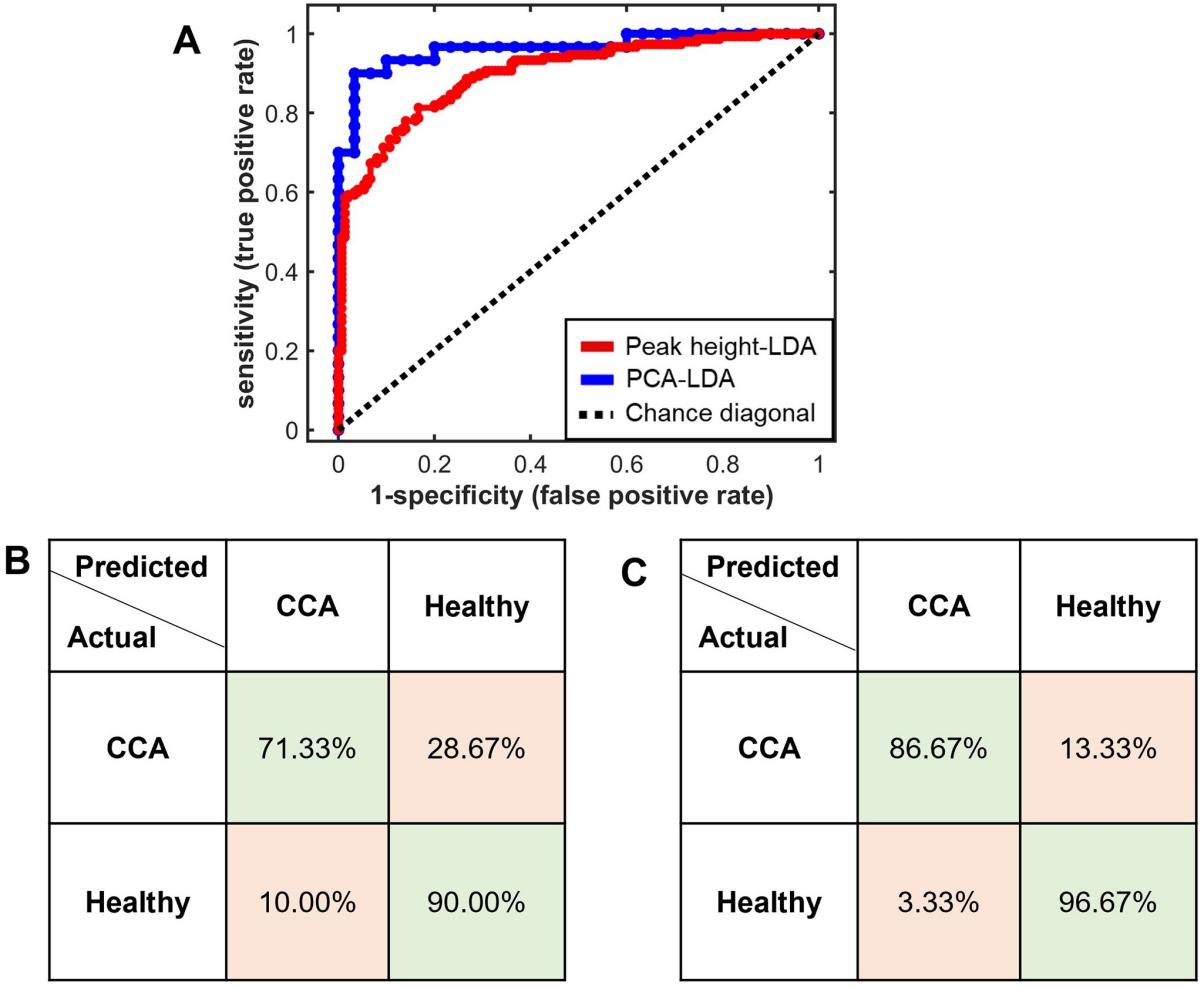
Cross-validated ROC curves and confusion matrices for classification between CCA and healthy groups. (A) The integrated area under the ROC curves was 0.963 for the PCA-LDA analysis and 0.899 for the peak height analysis combined with the LDA approach. The confusion matrices for the classification performance between (B) the peak height-LDA model and (C) the PCA-LDA model as validated by a 5-fold cross validation method.

To validate the model classification performance and prevent potential model overfitting, a k-fold cross-validation approach was employed [56]. The k-fold cross-validation results (k = 5; Table 2) confirmed that the PCA-LDA method distinguished CCA from healthy groups more effectively than the peak height-LDA approach, as indicated by higher values for all diagnostic performance parameters. The cross-validated receiver operating characteristic (ROC) curves and confusion matrices were generated (Fig 6) to further evaluate and compare the diagnostic performances of the two methods. In PCA-LDA, 86.67% of CCA were correctly identified, 13.33% were incorrectly classified as healthy samples, while only 3.33% of the healthy group was misclassified. The performance of peak height-LDA was inferior, with 28.67% of CCA incorrectly identified as healthy. In other words, peak height-LDA had a higher false negative rate (FNR: 28.67%) and false positive rate (FPR: 10.00%) than the PCA-LDA method (FNR: 13.33%, FPR: 3.33%). A high FPR would be unfavorable for a diagnostic test because it could expose healthy individuals to unneeded therapy, whereas a high FNR could result in patients being left untreated. In addition, the PCA-LDA method had a higher integrated area under the ROC curve (0.963) than the peak height-LDA method (0.899). The closer the value of the area under the ROC curve is to one, the more accurate the classifier’s predictions [57]. Therefore, these cross-validated classification results demonstrated that although both diagnostic algorithms were able to discriminate between CCA and healthy groups, the PCA-LDA-based diagnostic algorithm that utilized several prominent spectral features present in serum Raman spectra provided a more effective diagnostic performance for cholangiocarcinoma detection than the peak height-LDA method.

**Table 2:** The diagnostic performance in classification between the CCA and healthy groups using peak height-LDA and PCA-LDA methods together with a k-fold cross validation approach (k = 5).

Analysis Method	Cross-validated diagnostic performance		
	Sensitivity	Specificity	Accuracy
Peak height-LDA	71.33	90.00	80.67
PCA-LDA	86.67	96.67	91.67

The combined Raman spectroscopy and PCA-LDA approach presented has shown promise as a rapid, label-free technique for cholangiocarcinoma detection and screening, as evidenced by its comparatively high sensitivity (86.67%) and specificity (96.67%) to other diagnostic techniques (Table 3). Due to the limitations of existing diagnostic methods, the diagnosis of CCA remains challenging, and there has been a growing demand for a low-cost, easy-to-use detection and diagnostic screening approach. Non-invasive imaging modalities, such as ultrasonography, computed tomography (CT), and magnetic resonance imaging (MRI), can offer relatively high diagnostic sensitivity and specificity for identifying CCA features and staging, but come at a high cost and necessitate the use of experienced medical personnel. Thus, these techniques can present a challenge in poor-resource settings. The experience level of radiologists could also compromise the accuracy of diagnosis and the frequency of misdiagnoses [58]. ERCP-based methods provide a definitive diagnosis of CCA through the collection of biliary tissue; however, the procedures are invasive, can be time-consuming, and may expose patients to procedure-related risks. Furthermore, despite being minimally invasive, the CA19-9 and CEA biomarker methods are not specific to CCA and may be present in other malignancies, such as colorectal and gastric cancers. The developed Raman spectroscopic technique can provide a minimally invasive and low-cost diagnostic tool for CCA screening, which can be used in conjunction with other diagnostic methods. However, the method has limitations that require further research before it can be applied effectively in a clinical setting.

**Table 3:** Comparison of current diagnostic methods for cholangiocarcinoma detection.

Technique	Method	Diagnostic performance			Limitation	Price
		Sensitivity (%)	Specificity (%)	Reference		
Ultrasonography	Contrast-enhanced ultrasound	97	88	[62]	Hilar and extrahepatic CCA are difficult to detect [63]. Limited ability to distinguish between intrahepatic CCA and hepatocellular carcinoma (HCC) [64]. Requires experienced medical experts to interpret imaging results.	Medium (approx. 100–500 US$)
	Ultrasound	87–96	86–91	[65, 66]		
Radiologic imaging	Computed tomography (CT)	39–81	54–95	[63]	Contrast materials may cause allergies and kidney toxicity. Requires experienced medical experts to interpret imaging results. Very expensive.	Very high(approx. >/ 3000 US$)
	Magnetic resonance imaging (MRI)	23–91	40–95	[63]		
Endoscopic approaches	ERCP^a^ brush cytology	45–56	89	[11, 67]	ERCP-based methods Invasive and may present an increased risk of procedure-related issues, including pancreatitis, cholangitis, and perforation [11, 12].	High (approx.1000-2000 US$)
	ERCP biopsy	67	96	[11, 67]		
	ERCP brushing & biopsy	47–86	97–100	[68]		
	Endoscopic ultrasound (EUS) & fine needle aspiration (FNA)	74–86	95–100	[67, 69]	EUS-FNA Small risks such as bleeding or infection. Bile duct strictures may result in poor quality of cytologic specimens [70].	
Blood biomarker	Carbohydrate antigen (CA) 19–9	40–70	50–80	[12, 71]	Non-specific to CCA; may present in pancreatic and colorectal cancers and nonmalignant conditions, such as thyroidal diseases [12, 72].	Medium (approx. 100–500 US$)
	Carcinoembryonic antigen (CEA)	33–84	33–100	[73]		
Raman spectroscopy-based technique	Raman spectroscopy combined with PCA-LDA	86.7	96.67	Present work	Did not differentiate cancer stages Unable to identify the CCA anatomical site of origin in the biliary tract	Low (approx. 10 US$)

^a^ Endoscopic Retrograde Cholangiopancreatography (ERCP).

The capability of the developed Raman technique in determining CCA stages and classifying its anatomical origin has not been investigated and confirmed in this study. A larger population would be required to study the classification of CCA’s disease stage and anatomical origins, as the majority (77%) of the CCA patient samples used in this study were of advanced disease stage. Since CCA was typically diagnosed after the disease had manifested [1, 59, 60], the recruitment of early-stage volunteers may be difficult. In addition, the intrinsically small cross-section of Raman scattering of biomolecules may pose a challenge for Raman detection of early-stage cancer samples with subtle cancer-induced biochemical alterations. Other Raman-related techniques, such as SERS, may be utilized to enhance the Raman signal and improve the diagnostic sensitivity for early-stage CCA detection.

Despite demonstrating a relatively high sensitivity and specificity for CCA detection, the current method was performed on a commercial benchtop Raman spectrometer with highly optimized optical properties (e.g., signal-to-noise ratio, spectral resolution and stability). However, CCA is highly prevalent in resource poor rural communities with limited access to healthcare [61]. To be able to implement this technique in remote and resource-constrained endemic regions, the measurements may need to be acquired with a portable Raman device that potentially has inferior optical performance, thereby compromising the overall diagnostic performance of the method. Thus, future research could focus on optimizing this method on a lower performance portable spectrometer or developing highly sensitive portable Raman spectrometer for use with this technique in order to increase its accessibility to patients in remote areas. Collectively, the Raman approach combined with machine learning offers a promising strategy as a simple, rapid, and effective diagnostic method that can be further implemented for CCA screening in a large population and may be able to complement the existing imaging modalities to improve the overall CCA diagnosis and prognosis outcomes.

## Conclusion

The study presented here showed that the Raman spectroscopy technique combined with the PCA-LDA analysis as a non-invasive and rapid diagnostic approach could analyze serum differences and distinguish between cholangiocarcinoma patients and healthy subjects. The empirical analysis of the Raman spectra revealed several Raman bands whose peak heights showed statistically significant differences between the CCA and healthy groups. Serum from the cholangiocarcinoma patients contained significantly higher levels of cholesterol, tryptophan, and amide III but lower levels of beta-carotene than the healthy subjects. These biomolecular components can be identified as Raman marker bands for cholangiocarcinoma detection from serum. A peak height analysis of these Raman marker bands combined with LDA generated diagnostic performances of 80.67% accuracy, 71.33% sensitivity, and 90.00% specificity in classifying between the CCA and healthy serum specimens, with an area under the ROC curve of 0.899. In comparison, the PCA-LDA technique provided better diagnostic performances of 91.67% accuracy, 86.67% sensitivity, and 96.67% specificity for cholangiocarcinoma detection, with a higher ROC area under the curve of 0.963. Thus, Raman spectroscopy combined with PCA–LDA provided a promising tool for cholangiocarcinoma serum detection and screening. In future work, the capability of this technique in discrimination of CCA stages and anatomical sites of origin will be further explored.

## Supporting information

S1 Fig Loading spectra of the PCA analysis. (A) Loadings of 12 PCs acquired from the PCA analysis of the total data set (150 measurements for each group). (B) Loadings of 7 PCs acquired from the averaged data set (averaged from five independent measurements of each specimen). Significant PCs (p-value ≤ 0.05) are indicated by *.
(TIF)
