# Supplementary figures and images for: Rapid label-free detection of cholangiocarcinoma from human serum using Raman spectroscopy
Source: PLoS One. 2022 Oct 13;17(10):e0275362. doi: 10.1371/journal.pone.0275362 (PMC9562168; doi:10.1371/journal.pone.0275362)

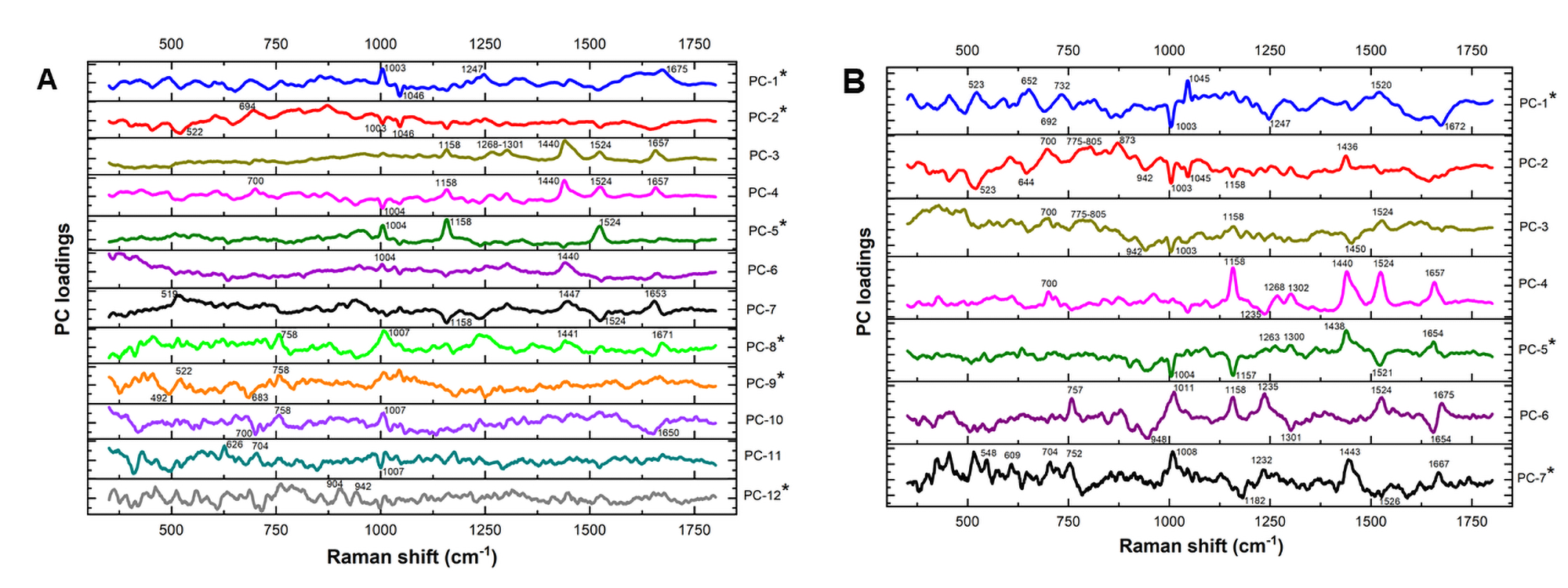

Supplement: S1 Fig — (A) Loadings of 12 PCs acquired from the PCA analysis of the total data set (150 measurements for each group). (B) Loadings of 7 PCs acquired from the averaged data set (averaged from five independent measurements of each specimen). Significant PCs (p-value ≤ 0.05) are indicated by *. (TIF) [file pone.0275362.s001.tif]
